# Impact of severe periodontitis and diabetes mellitus on recurrent cardiovascular outcomes

**DOI:** 10.1002/jper.70086

**Published:** 2026-03-06

**Authors:** Stefan Reichert, Axel Schlitt, Selina Rehm, Susanne Schulz

**Affiliations:** ^1^ Department of Operative Dentistry and Periodontology Martin Luther University Halle‐Wittenberg Halle Germany; ^2^ German Pension Insurance Braunschweig Hanover Laatzen Germany; ^3^ Medical Faculty Martin Luther University Halle‐Wittenberg Halle Germany

**Keywords:** cardiovascular disease, diabetes mellitus, periodontitis, recurrent cardiovascular events

## Abstract

**Background:**

This post hoc analysis of the Periodontitis and Coronary Heart Disease study (ClinicalTrials.gov Identifier: NCT01045070) aimed to determine the association between diabetes and recurrent cardiovascular events (CVEs) in patients with cardiovascular disease (CVD) and severe periodontitis. Another objective was to examine the link between diabetes and severe periodontitis.

**Methods:**

A cohort of 1,002 stationary patients with angiographically proven CVD was included. The patients were examined for the prevalence of diabetes (severity levels: diet, oral antidiabetic drugs, and insulin) and severe periodontitis (≥30% of teeth with ≥5 mm of proximal attachment loss). Recurrent CVEs were summarized as a combined endpoint (myocardial infarction, stroke/transient ischemic attack, cardiovascular death, and stroke‐related death). After a 10‐year follow‐up period, survival analyses were carried out. Hazard ratios (HRs) were adjusted for known cardiac risk factors using Cox regression.

**Results:**

A total of 792 patients completed follow‐up. The overall incidence of the combined endpoint was 42.8%. The highest HR for CVEs was observed in patients with both diabetes and severe periodontitis (HR = 2.19, 95% confidence interval [CI] 1.59‐3.02). HRs were lower in subjects with only periodontitis (HR = 1.52, 95% CI = 1.14‐2.04) or only diabetes (HR = 1.88, 95% CI = ∖1.35‐2.63). Patients with diabetes who took drugs or required insulin were more likely to have severe periodontitis than patients without diabetes.

**Conclusion:**

Patients with diabetes or severe periodontitis were at an increased risk of recurrent CVEs. An association was also found between diabetes and severe periodontitis prevalence.

**Clinical trial registration:**

ClinicalTrials.gov Identifier: NCT01045070

**Plain language summary:**

Periodontitis is a serious gum disease that damages the tissue that supports teeth. In its final stage, it can result in tooth loss. Furthermore, studies have shown an association between periodontitis and cardiovascular disease (CVD) and diabetes mellitus. The objective of this study was to investigate the relationship between diabetes and recurrent cardiovascular events (CVEs) in patients with CVD and severe periodontitis. Another objective was to examine the link between diabetes and severe periodontitis. Thus, 1,002 inpatients with CVD were examined for periodontal disease and other medical conditions, such as diabetes mellitus, at the time of the baseline examination. Ten years later, questionnaires and patient records were used to determine whether new CVEs had occurred. CVEs include myocardial infarction, stroke/transient ischemic attack, cardiovascular death, and death caused by stroke. The results showed that severe periodontitis and diabetes mellitus, especially when the two conditions occurred together, increased the risk of CVEs. Furthermore, a higher percentage of patients with diabetes also suffered from severe periodontitis. Further studies should clarify whether periodontal and antidiabetic therapies reduce the risk of CVEs.

## 1 INTRODUCTION

Diabetes is categorized as a noncommunicable disease, with a reported global prevalence of 9.3% (463 million people).^1^ The primary symptom is chronic hyperglycemia, which results from impaired insulin secretion and/or insulin resistance.^2^ Chronic hyperglycemia has been shown to promote the development of micro‐ and macroangiopathies and cause diabetes‐related complications. These complications include retinopathy and maculopathy, neuropathy, nephropathy, and atherosclerotic diseases such as peripheral arterial disease (PAD) and cardiovascular disease (CVD).^3^

Numerous studies have also demonstrated an association between periodontitis and other diseases. These include the initial manifestation of CVD ^4^, ^5^, ^6^, ^7^ and recurrent cardiovascular events (CVEs) among patients with preexisting CVD.^8^, ^9^, ^10^, ^11^ Periodontitis is characterized by microbial‐associated, host‐mediated inflammation that results in the loss of periodontal attachment.^12^ In 2021, more than one billion people (1066.95 million; 95% uncertainty interval [UI]: 896.55‐1234.84) worldwide were affected by severe periodontitis, corresponding to a global age‐standardized prevalence of 12.50%.^13^ The relationship between diabetes mellitus and severe periodontitis appears to be bidirectional. Some studies have focused on the link between diabetes and periodontitis. For example, gingivitis, which can progress to periodontitis under certain conditions, demonstrated accelerated progression, higher prevalence, and more severe manifestations in patients with diabetes than in nondiabetic patients.^14^, ^15^ Additionally, periodontitis was more prevalent among diabetics than nondiabetics and occurred at an earlier age. Periodontal bone and attachment loss were more pronounced, and periodontal destruction progressed more rapidly.^16^, ^17^ Individuals with diabetes who fail to regulate their blood sugar levels adequately are three times more likely to develop periodontitis.^18^ Consequently, the risk of developing and progressing periodontitis increases, particularly among diabetics whose blood sugar levels are not adequately controlled.

Other studies have observed an association between periodontitis and diabetes mellitus. For example, a significant association was found between severe periodontitis and elevated serum levels of HbA1c.^19^ Individuals with periodontitis are at a higher risk of developing type 2 diabetes.^20^ Additionally, a direct link has been established between the severity of periodontitis and diabetes complications, including retinopathy, neuropathy, nephropathy, cardiovascular complications, and mortality.^21^

Due to the described links between periodontitis, diabetes, and CVD, the first goal of our study was to investigate whether diabetes is a risk factor for recurrent CVEs in patients with severe periodontitis and CVD. A secondary objective was to verify the association between diabetes mellitus and the prevalence of severe periodontitis. To this end, we conducted a post hoc analysis of the “Periodontitis and Coronary Heart Disease” study (ClinicalTrials.gov Identifier: NCT01045070). We defined recurrent CVEs as myocardial infarction (MI), transient ischemic attack (TIA), stroke, cardiovascular death, or fatal stroke. Patients were observed for 10 years following their initial inpatient treatment at the Heart Center of the University Hospital Halle (Saale).

## 2 MATERIALS AND METHODS

### 2.1 Study population

The investigations were carried out in accordance with the ethical guidelines of the “Declaration of Helsinki” and its amendments in Tokyo and Venice. The study was approved by the ethics committee of the Martin Luther University Halle‐Wittenberg (protocol dated July 18, 2007; supplementary protocol dated April 25, 2013).

**FIGURE 1:**
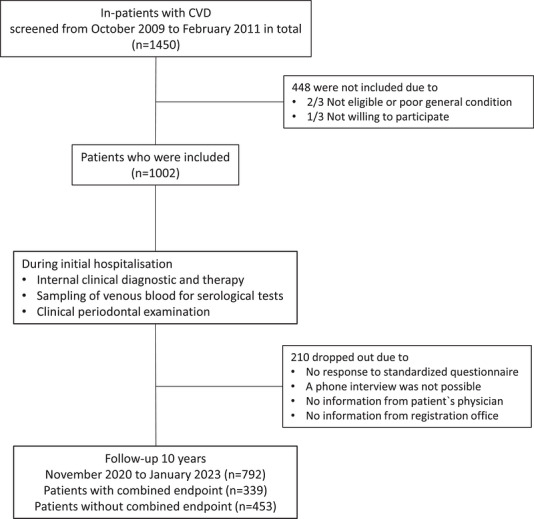
Study design and flow.

The present study is a longitudinal observational study. It was designed and conducted in accordance with the STROBE checklist for observational studies ^22^ (Figure 1). A total of 1,002 consecutive Caucasian patients originating from central Germany who suffered from CVD were enrolled in a prospective study at the Heart Center of the University Hospital Halle (Saale). Recruitment took place from October 2009 to February 2011. The reasons for hospitalization are outlined in Table 1. Inclusion criteria encompassed individuals aged 18 years and over with a confirmed history of CVD. This diagnosis was established by the presence of 50% stenosis of a major coronary artery, as evidenced by coronary angiography or percutaneous coronary intervention (PCI) or, alternatively, coronary artery bypass graft (CABG) surgery. Additionally, having at least four teeth was a mandatory inclusion criterion. Exclusion criteria included subjects who were unable to provide written informed consent, those who had undergone periodontal treatment (surgical or nonsurgical within the previous six months), and those who had received antibiotic therapy within the previous three months prior to the examination. Cases of pregnancy were also excluded from the study. Patients were also excluded if they had any disease or disorder that, in the investigators’ opinion, precluded participation in this clinical trial, such as current drug or alcohol abuse.

**TABLE 1:** Reasons for hospitalization of patients admitted at the beginning of the study

Reasons for hospitalization	Frequency	Percentages
Stable angina pectoris	48	4.8
Instable angina pectoris	243	24.3
Non‐ST‐elevation myocardial infarction (NSTEMI)	98	9.8
ST‐elevation myocardial infarction (STEMI)	62	6.2
Implantation of a pacemaker or implantable cardioverter defibrillator (PM/ICD).	34	3.4
Heart failure	61	6.1
Elective cardiac catheterization	89	8.9
Hypertensive crisis	26	2.6
Atrial fibrillation	48	4.8
Other arrhythmias	36	3.6
Pulmonary embolism	3	.3
Valvular heart disease	31	3.1
Aortocoronary bypass surgery	126	12.6
Other	97	9.7

The following basic variables were collected: age; body mass index (BMI, calculated as body mass [kg] divided by body length [m^2^]); current smoking status; and current diseases, such as diabetes mellitus, hypertension, and dyslipidemia. A patient was diagnosed with diabetes mellitus if it was noted in their medical history and/or if they were receiving dietary or antidiabetic therapy in the hospital or had a fasting blood glucose level of at least 7 mmol/L. The cohort of patients with diabetes mellitus was divided into three groups. The first group received insulin, the second group received oral antidiabetic medication, and the third group received dietary recommendations. Dyslipoproteinemia was diagnosed based on a prior diagnosis, therapy with lipid‐lowering agents, or a fasting cholesterol level of > 5.2 mmol/L or low‐density lipoprotein (LDL) cholesterol of > 3.9 mmol/L. Arterial hypertension was defined as hypertension diagnosed prior to current admission, use of antihypertensive medication, or a blood pressure measurement of > 140/90 mmHg.

A classification system developed specifically for risk factor analysis was chosen for the diagnosis of severe periodontitis.^23^ Severe periodontitis was diagnosed when at least 30% of present teeth had proximal attachment loss of at least 5 mm. Clinical attachment loss (CAL) is defined as the distance between the enamel‐cementum junction of a tooth and the apical stop of the probe at a probing pressure of 0.2 N. Measurements were taken using a pressure‐calibrated periodontal probe*. To assess periodontitis activity, we also determined the percentage of teeth that were bleeding on probing (BOP).^24^

### 2.2 Follow‐up

The 10‐year follow‐up surveys were conducted between November 2020 and January 2023. For the present study, we evaluated subsequent cardiovascular events in participants who were readmitted to the clinic during the follow‐up period using the electronic patient record. For the remaining patients, self‐reported information was used instead. These patients were given a standardized questionnaire (see the supplementary material) with a prepaid, preaddressed envelope for returning it. If the participant's address was invalid, we contacted the relevant registration authorities to ascertain the participant's current address. If the study participant did not respond within eight weeks, a subsequent questionnaire was sent. If the second questionnaire was also unanswered, we attempted to interview the patient or their relatives via telephone. If the participant died during the interim period, we contacted the relevant health authorities to determine the date and cause of death. If further information could not be obtained from the aforementioned subjects, civil registration offices were contacted for details regarding the subjects' current addresses or dates of death. Data collected during the follow‐up period were used to calculate the incidence of new CVEs. The primary outcome of interest was defined as a composite endpoint incorporating MI, stroke/ TIA, cardiac death, and stroke‐related death.

### 2.3 Statistical analyses

#### 2.3.1 Power analysis

In patients with a documented history of CVD, the estimated 5‐year risk of recurrent CVEs was found to be approximately 20% higher than in patients without a history of CVD, after accounting for the standard risk factors.^25^ In 2005, 34% of senior citizens in Germany aged 65 to 74 were diagnosed with severe periodontitis.^26^ According to data from the Robert Koch Institute, the prevalence of diabetes mellitus was 20.9%.^27^ The power analysis was based on the following assumptions: First, we hypothesized that the combined endpoint will be observed in at least of 15% of subjects. We estimated that approximately 50% of subjects have severe periodontitis, while 20% are diagnosed with diabetes mellitus. Furthermore, we hypothesized that the probability of the combined endpoint occurring in individuals with severe periodontitis and/or diabetes is at most twice as high. After enrolling 800 patients, the 95% confidence interval of the relative risk (estimated value of 2) would have a lower limit of 1.4. This would provide a sufficiently accurate estimate of the association between severe periodontitis and/or diabetes and the combined endpoint. Considering the anticipated attrition rate of about 20%, 1,002 subjects were initially enrolled in the study.

#### 2.3.2 Univariate and multivariate comparisons

Statistical analyses were performed using commercially available software†. Values of p ≤ 0.05 were considered to be significant. The normality of the demographic and clinical metric data was established using the Kolmogorov–Smirnov and Shapiro–Wilk tests. Since all metric values were found to be non‐normal, they were presented as medians and 25th/75th percentiles. The Mann–Whitney U test was used for the statistical analysis. Categorical variables were expressed as percentages. The chi‐squared test was employed for the purpose of comparison.

Univariate survival analyses were conducted using the Kaplan‐Meier method with a log‐rank test. To assess the independence of severe periodontitis and/or diabetes mellitus as risk factors for recurrent CVEs, a Cox regression analysis was performed, with adjustments made for other risk factors including age, male sex, BMI, BOP, PAD, hypertension, dyslipidemia, current smoking, and lipid‐lowering agent intake.

## 3 RESULTS

### 3.1 Patient characteristics

A total of 1,002 consecutive patients were enrolled prospectively. After an average follow‐up period of 378.3 weeks (± 214.8), data were available for 792 patients. This corresponds to a dropout rate of 21% (Figure 1).

The median age of the overall cohort was 69 years. Among the participants, 73.5% were male, and 10.4% were active smokers at the time of enrollment. The prevalence of severe periodontitis and diabetes mellitus was 47.9% and 35.6%, respectively. Among the CVD patients, 18.7% had both severe periodontitis and diabetes mellitus (Table 2). The overall incidence of the combined endpoint was 42.8% (11.2% MI, 7.5% stroke or TIA, 22.6% cardiovascular death, and 1.5% fatal stroke).

**TABLE 2:** Baseline characteristics of the investigated patients with CVD in dependence of the incidence of the combined endpoint

Variables	All patients (n = 792)	No event (n = 453)	Event (n = 339)	Pearson Chi^2^ coefficient	*p*‐values
Age, median (25^th^/75^th^ percentiles)	69.20 (60.68/74.83)	68.20 (59.34/72.87)	70.91 (62.70/77.42)	‐	**<0.001**
Male (% individuals)	73.5	71.3	76.4	2.59	0.108
BMI, kg/$m^{2}$, median (25^th^/75^th^ percentiles)	28.09 (25.34/30.72)	28.06 (25.31/30.86)	28.23 (25.35/30.68)	‐	0.691
Current smoker (% individuals)	10.4	11.5	8.8	1.44	0.229
Diabetes mellitus (% individuals)	35.6	29.4	44.0	18.01	**<0.001**
Insulin dependent	18.2	11.5	27.1		
Oral drugs	14.1	14.8	13.3		
Dietic treatment	3.3	3.1	3.5	33.0	**<0.001**
Hypertension (% individuals)	87.6	86.1	89.7	2.30	0.130
Dyslipidemia (% individuals)	59.0	59.2	58.7	0.02	0.897
Peripheral arterial disease (% individuals)	10.2	7.3	14.2	9.98	**0.002**
Intake of lipid lowering drugs (% individuals)	88.3	88.3	88.2	0.002	0.966
Severe periodontitis^*^(% individuals)	47.9	43.3	54.0	8.92	**0.003**
Bleeding on probing (% teeth), median (25th/75th percentiles)	5.6 (1.9/12.5)	5.0 (1.7/10.9)	6.3 (2.3/12.5)		**0.013**
% Individuals					
No severe periodontitis, no diabetes	35.2	41.9	26.3		
Severe periodontitis, no diabetes	29.2	28.7	29.8		
Diabetes, no severe periodontitis	16.9	14.8	19.8		
Diabetes, severe periodontitis	18.7	14.6	24.2	11.8	**<0.001**

*Note*: Statistical comparisons between patients with event and no event were made by Chi‐square test for categorial variables and by Mann–Whitney U‐test for continous variables. Significant p‐values are highlighted in bold.

Abbreviation: BMI, body mass index.

* Proximal attachment loss of ≥5 mm in ≥30% of teeth present.

### 3.2 Demographic and clinical data relating to the combined endpoint

Patients who had experienced a recurrent CVE were older (70.9 versus 68.2 years), had a higher BOP (6.3% versus 5.0%), and were more likely to have severe periodontitis (54% versus 43.3%) or diabetes mellitus (44% versus 29.4%). Another notable finding was the increased prevalence of patients with both periodontitis and diabetes mellitus (24.2% versus 14.6%). Twice as many patients in the group with events were found to have PAD (14.2% versus 7.3%) (Table 2).

### 3.3 Association between severe periodontitis and diabetes

**FIGURE 2:**
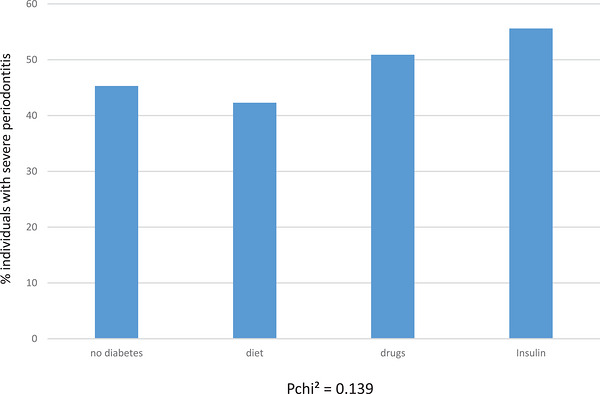
Prevalence of severe periodontitis in dependence of severity of diabetes mellitus.

At the inception of the study, the prevalence of severe periodontitis was higher among diabetics treated with medication or insulin than among those advised to follow a diet only. The prevalence of periodontitis in patients without diabetes fell between the diet‐ and medication‐controlled diabetes groups (Figure 2).

### 3.4 Survival analyses

#### 3.4.1 Univariate analyses

**FIGURE 3:**
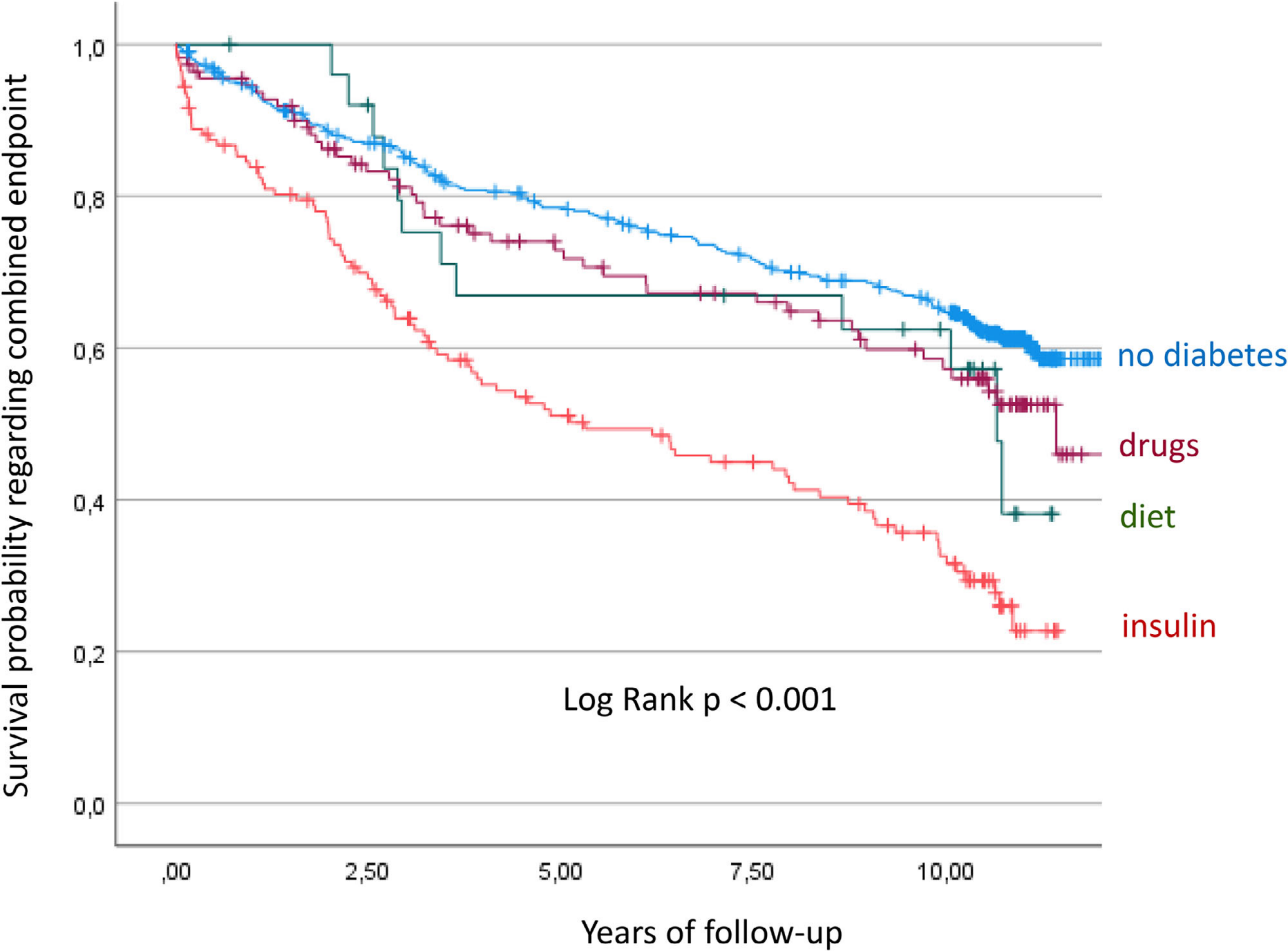
The Kaplan–Meier plot shows the combined endpoint (stroke, transient ischemic attack [TIA], myocardial infarction, and cardiovascular death) according to the severity of diabetes mellitus. Statistical comparisons were made using the log‐rank test.

Patients diagnosed with insulin‐dependent diabetes mellitus (27.1% CVEs, unadjusted HR = 2.7) had a higher risk of developing the combined endpoint than patients whose diabetes treatment included dietary interventions (3.5% CVEs, unadjusted HR = 1.4) or medication (13.3% CVEs, unadjusted HR = 1.3) (Figure 3).

**FIGURE 4:**
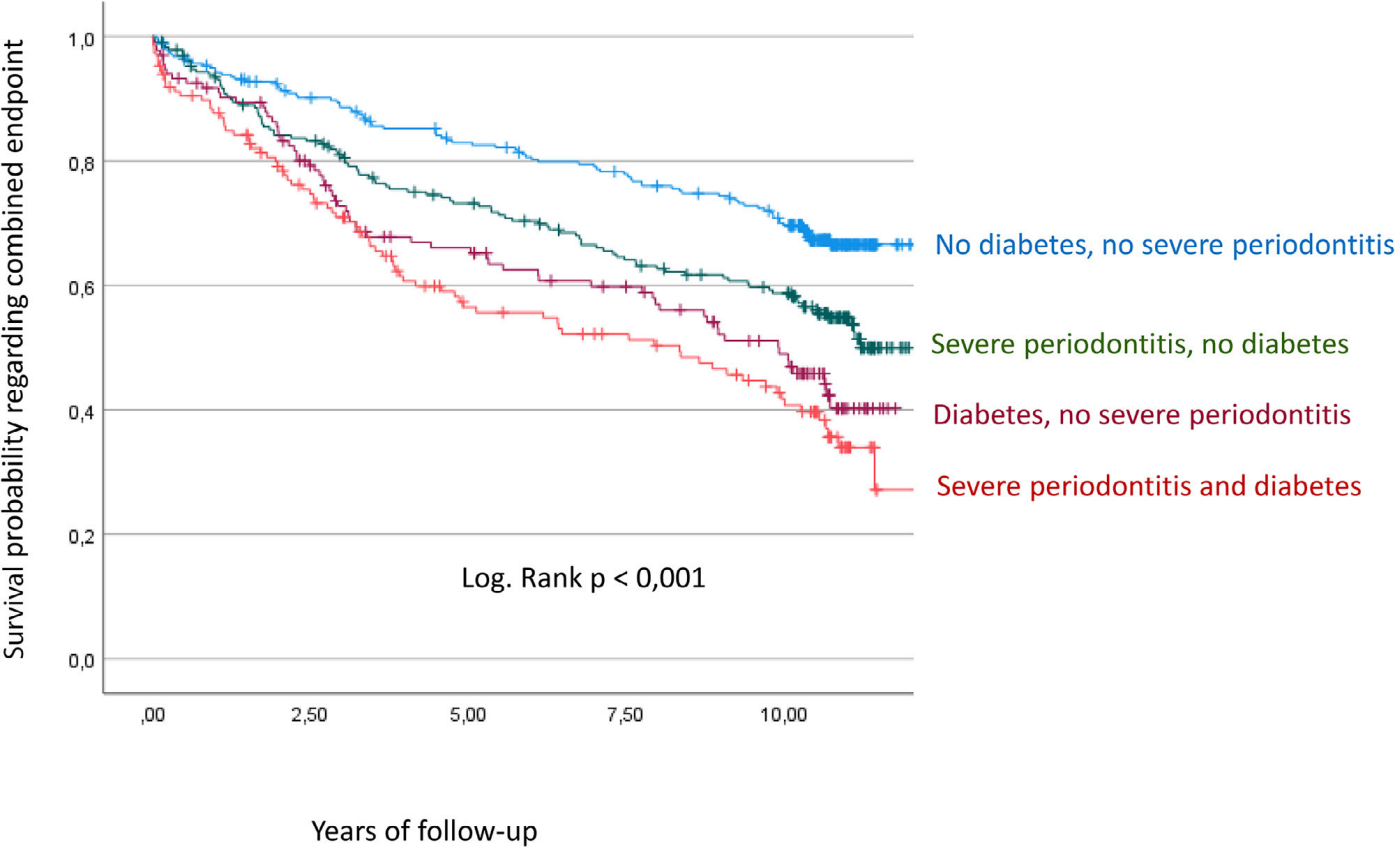
The Kaplan–Meier plot shows the combined endpoint (stroke, transient ischemic attack [TIA], myocardial infarction, cardiovascular death, and death caused by stroke) according to the prevalence of diabetes or severe periodontitis alone, or diabetes plus severe periodontitis, compared to patients who have neither. Statistical comparisons were made using the log‐rank test.

Patients with CVD who did not have diabetes or severe periodontitis had a lower risk of new CVEs than patients who had either or both conditions (Figure 4). The unadjusted HR for CVEs was 1.6 for patients with CVD and periodontitis, 2.1 for patients with CVD and diabetes, and 2.6 for patients with both.

#### 3.4.2 Multivariate analyses

The results of the univariate analysis were verified. When adjusted for confounding variables, including age, sex, BMI, smoking, BOP, hypertension, PAD, dyslipidemia, and lipid‐lowering medication, the adjusted HR was highest in patients with both diabetes and severe periodontitis (HR = 2.19). This was followed by patients with diabetes but without severe periodontitis (HR = 1.88) and patients with severe periodontitis but without diabetes (HR = 1.52). Age, male sex, and PAD were identified as additional risk factors for the combined endpoint (Table 3).

**TABLE 3:** Adjusted hazard ratios for diabetes and severe periodontitis in isolation, as well as for the combination of both factors for the incidence of the combined endpoint (stroke/TIA, myocardial infarction, cardiovascular death, death as consequence of stroke) calculated with cox regression

	Hazard ratio	95.0% CI		
Confounding variables		**Lower**	**Upper**	*p*‐value
Age	1.04	1.03	1.05	**<0.001**
Male sex	1.35	0.57	0.95	**0.022**
Body mass index	0.99	0.97	1.02	0.705
Current smoking	1.23	0.81	1.85	0.329
Peripheral arterial disease	1.99	1.46	2.73	**<0.001**
Hypertension	1.14	0.79	1.66	0.473
Dyslipoproteinaemia	0.91	0.72	1.15	0.442
Intake of lipid lowering drugs	0.9	0.64	1.27	0.553
Bleeding after probing	1.0	0.99	1.01	0.432
Severe periodontitis, no diabetes	1.52	1.14	2.04	**0.005**
Diabetes, no severe periodontitis	1.88	1.35	2.63	**<0.001**
Diabetes and severe periodontitis	2.19	1.59	3.02	**<0.001**

Note: Significant p‐values are highlighted in bold.

Abbreviation: CI, confidence interval; TIA, transient ischemic attack.

## 4 DISCUSSION

The study showed that recurrent CVEs develop more frequently in patients with CVD who were diagnosed with diabetes mellitus or severe periodontitis at the time of the baseline examination, particularly if both conditions were present simultaneously. BOP was only associated with CVEs in the univariate comparison and was not associated with them in the multivariate survival model. Insulin‐dependent diabetics had a higher risk of recurrent CVEs than diabetics who took oral antidiabetic drugs or were recommended a special diet. The study also demonstrated a link between the severity of diabetes and the prevalence of severe periodontitis. The prevalence of periodontitis was higher among diabetics who required insulin than among subjects who took oral antidiabetic drugs, relied solely on a special diet, or were not diabetic.

Two additional studies ^28^, ^29^ support the results of our study. One prospective longitudinal study ^28^ involving 628 Pima Indians with type 2 diabetes aged 35 years or older investigated the influence of periodontitis on overall and CVD‐specific mortality. Arizona Pima Indians have the highest prevalence of type 2 diabetes mellitus worldwide.^30^ The severity of periodontitis was categorized as absent, mild, moderate, or severe based on panoramic X‐rays and clinical dental examinations. After an 11‐year follow‐up period, the researchers adjusted for age, sex, diabetes duration, HbA1c level, macroalbuminuria, BMI, serum cholesterol concentration, high blood pressure, electrocardiographic abnormalities, and current smoking in a proportional hazards model. They found that subjects with severe periodontitis had a 3.2‐fold higher risk (95% confidence interval [CI]: 1.1‐9.3) of cardiorenal mortality (combined ischemic heart disease and diabetic nephropathy) than the reference group, consisting of subjects with no, mild, or moderate periodontitis.

The second study ^29^ aimed to determine whether periodontal infections significantly influence the increased risk of carotid intima‐media thickness (IMT) and advanced atheromatous lesions posed by diabetes. Cross‐sectional data from 6,048 individuals aged 52 to 74 years were obtained from the Atherosclerosis Risk in Communities (ARIC) Study. Participants without diabetes (n = 5,257) were compared to those with diabetes (n = 791). The dependent variables were IMT thickness over 1 mm, acoustic shadowing, and prevalent coronary heart disease (CHD). Individuals with diabetes and severe periodontitis were significantly more likely than individuals without diabetes or periodontitis to have an IMT greater than 1 mm (adjusted odds ratio [OR] = 2.2; 95% confidence interval [CI] = 1.4‐3.5), acoustic shadowing (adjusted OR = 2.5; 95% CI = 1.3‐4.6), and CHD (adjusted OR = 2.6; 95% CI = 1.6‐4.2).

In our study, periodontal status and diabetes mellitus were assessed only at baseline, not at follow‐up. Additionally, no data on blood glucose levels are available. Therefore, we can only make assumptions about the pathophysiological basis of the observed associations. Periodontitis has been associated with elevated HbA1c levels in both diabetic patients ^19^ and in individuals without diabetes.^31^, ^32^ The HbA1c value depends on the stage of periodontitis.^33^ Prolonged exposure of cells to high glucose concentrations leads to intracellular hyperglycemia, particularly in endothelial vascular cells with low glucose transport rates. These cells then become primary targets of hyperglycemic damage, due to increased reactive oxygen species production and altered gene expression. These changes can lead to pathological alterations that trigger microvascular complications.^34^ Furthermore, periodontal bacteria and their toxins directly influence blood vessels, and the systemic inflammatory response caused by periodontitis is also likely to influence cardiovascular outcomes.^35^ Bacteremia can be caused by everyday activities such as tooth brushing ^36^ and professional interventions such as scaling and root planing.^37^ It occurs more frequently, lasts longer, and involves more virulent bacteria in patients with periodontitis.^38^ Furthermore, viable oral bacteria, including *Porphyromonas gingivalis* (*P.g*.) and *Aggregatibacter actinomycetemcomitans* (*A.a*.), have been found in atherosclerotic lesions.^39^, ^40^ Evidence that periodontal pathogens can promote atheroma formation has been obtained using different animal models.^41^, ^42^ Additionally, higher levels of C‐reactive protein ^43^ and interleukin (IL) ‐6^44^ have been observed in patients with severe periodontitis compared to healthy controls. Both are inflammatory mediators associated with the pathophysiology of atherosclerosis.

Our data also revealed a direct association between diabetes mellitus and the prevalence of severe periodontitis (Figure 2). This result was confirmed by another large study conducted over 5 years in Germany. The authors found that people with uncontrolled type 2 diabetes experienced more severe progression of periodontitis than those with controlled diabetes or no diabetes.^31^ One potential explanation for the increased susceptibility to periodontitis among individuals with diabetes may be the functional abnormalities of polymorphonuclear neutrophil granulocytes (PMNs) caused by diabetes. PMNs play a crucial role in maintaining periodontal health.^45^ Furthermore, in diabetic patients, advanced glycation end products (AGEs) can accumulate in periodontal tissues. Interactions between AGEs and their receptors (RAGE) activate the production of proinflammatory cytokines. These, in turn, activate osteoclasts and collagenases/matrix metalloproteinases, which can ultimately result in the destruction of periodontal structures.^46^

Based on the findings of this study, it is evidently important for patients with diabetes to undergo periodontal examination and treatment as needed. Our study could not examine these kinds of interventions and their influence on recurrent CVEs. Furthermore, there are currently no convincing prospective studies proving that treating periodontitis in diabetic patients actually improves cardiovascular outcomes. However, nonsurgical periodontal treatment could reduce HbA1c levels in patients who suffered from both diabetes and periodontitis.^47^, ^48^ The extent of the reported HbA1C reductions was 0.43% (95% CI: 0.28%‐0.59%) after 3‐4 months following periodontal therapy, 0.3% after 6 months (95% CI: 0.08%‐0.52%), and 0.5% after 12 months (95% CI: 0.45%‐0.55%).^47^

### 4.1 Limitations of the study

This was a monocentric study, focusing primarily on patients from Central Germany. Applying these results to other (ethnic) groups should therefore be done with caution. As the study was started before 2017, the new classification of periodontal diseases could not yet be implemented. In the present study, severe periodontitis was defined as approximal CAL of 5 mm or more in at least 30% of teeth. According to the new classification, this would correspond to stage III or IV. Diabetes and severe periodontitis could only be diagnosed at the beginning of the study and not at the follow‐up stage. Changes in glycemic status or periodontal conditions that may have occurred during the long observation period could not be considered concerning their potential influence on recurrent CVEs.

The different CVD types that led to hospitalization may influence the incidence of the combined endpoint. In 2010, the American Heart Association defined a new concept of cardiovascular health to promote a paradigm shift from a focus on treating disease to promoting and maintaining health across the lifespan for populations and individuals. The components of Life's Essential 8 include diet, physical activity, nicotine exposure, sleep health, body mass index, blood lipids, blood glucose, and blood pressure.^49^ Because the Life's Essential 8 were published after the present study was designed, some of these factors, such as diet, physical activity, and sleep health, were not included as covariates in this study.

## 5 CONCLUSION

Severe periodontitis and diabetes were associated with an increased risk of recurrent CVEs in patients with CVD. The level of risk is higher than for diabetes or periodontitis alone. The prevalence of severe periodontitis was associated with the severity of diabetes.

## AUTHOR CONTRIBUTIONS

**Stefan Reichert**: Conceptualization (equal); methodology; project administration; resources; supervision; validation; writing—original draft preparation; writing—review and editing. **Axel Schlitt**: Conceptualization (lead); methodology; project administration; resources; supervision; funding acquisition. **Selina Rehm**: Data curation; formal analysis; investigation; visualization. **Susanne Schulz**: Conceptualization (equal); methodology; formal analysis; validation, writing—review and editing.

## CONFLICT OF INTEREST STATEMENT

The authors have no conflicts of interest to disclose.

## Data Availability

The data that support the findings of this study are available upon request from the corresponding author. The data are not publicly available due to privacy or ethical restrictions.

## Footnotes

* UNC 15 0.2 N Aesculp, Tuttlingen, Germany

† SPSS v.25.0 package, IBM, Chicago, IL
